# Hypothesis: Coupling between Resorption and Formation in Cancellous bone Remodeling is a Mechanically Controlled Event

**DOI:** 10.3389/fendo.2015.00082

**Published:** 2015-05-20

**Authors:** Reinhold G. Erben

**Affiliations:** ^1^Institute of Physiology, Pathophysiology and Biophysics, Department of Biomedical Sciences, University of Veterinary Medicine Vienna, Vienna, Austria

**Keywords:** bone remodeling, coupling, bone formation, bone resorption, disuse

## Abstract

Coupling is the process that links bone resorption to formation in a temporally and spatially coordinated manner within the remodeling cycle. In order to maintain skeletal integrity, it is of crucial importance that the amount of bone resorbed matches the amount of newly formed bone in each remodeling site. Although a number of different explanatory models have been developed, the mechanisms that couple bone resorption and formation in bone remodeling are still a matter of controversy. Here, I propose a model in which coupling is achieved by biomechanical strain sensed by osteocytes within the newly built bone package. In this model, the resorption cavity created by osteoclasts results in mechanical weakening of the structural element, and, thus, in increased strain under constant loading conditions. Subsequent bone formation is initiated by strain-sensitive osteocytes in the underlying bone matrix. After osteoblastic bone formation has started, the newly built osteocyte–osteoblast network detects strain. Once the mechanical strain within the newly built bone structural unit falls below a certain threshold, bone formation stops. In this biomechanical strain-driven model, osteoblasts do not need to “know” how much bone was previously resorbed in a given site. In addition, this model does not require the transfer of any information from bone-resorbing osteoclasts to bone-forming osteoblasts, because biomechanical strain “guides” osteoblasts through their job of re-filling the resorption cavity.

Bone remodeling is a cyclical renewal process in which, after activation, a quantum of bone is first resorbed by osteoclasts. Thereafter, osteoblasts fill the resorption cavity with new bone in the same place. In contrast, bone formation and bone resorption are not coupled, and occur independently from each other during bone modeling, resulting in resorption or formation drifts which alter bone structure at the microscopic or macroscopic level.

Bone remodeling and bone modeling can both be found in cancellous and cortical bone of higher mammals. In a growing mammalian skeleton, cancellous bone turnover is dominated by modeling, whereas remodeling is the major turnover activity in a mature skeleton (1). In cortical bone, intracortical bone remodeling leaves behind typical microanatomical structures, namely Haversian canals or osteons. In intracortical bone remodeling, osteoclasts and osteoblasts are organized in a complex structure, the so-called basic multicellular unit (BMU). BMUs consist of a cutting cone of osteoclasts, followed by a closing cone lined by osteoblasts, together with connective tissue, blood vessels, and nerves (2). In cancellous bone, it is not entirely clear whether BMUs exist as distinct entities, because the length of the reversal phase appears to be quite variable, at least in postmenopausal osteoporosis (3, 4). The reversal phase is the phase between the end of resorption and the beginning of formation in the remodeling cycle.

It is currently thought that remodeling can be initiated by either stochastic, hormone-driven, or targeted, microdamage-driven, mechanisms. Stochastic remodeling is believed to be under endocrine control, with sex steroids and parathyroid hormone being the main endocrine determinants of bone turnover (5, 6). The purpose of targeted remodeling is to remove microdamage within the bone matrix. However, this distinction between stochastic and targeted remodeling may be arbitrary, because there is currently no proof that both mechanisms operate really independently. In any case, the initial event for initiation of osteoclastic bone resorption in cancellous bone remodeling is likely detachment of bone lining cells from the bone surface, at least in humans (7). Bone lining cells are flat, osteoblast-derived cells covering all quiescent bone surfaces. By detachment of bone lining cells from the bone surface, a canopy is formed under which blood-borne osteoclasts can attach to the bone surface and can start to resorb bone (7). Bone lining cells are able to receive information from osteocytes within the remodeling unit, because they are in contact with underlying osteocytes via gap junctions (8). Osteocytes appear to have a pivotal function not only for detection of microdamage within bone (9) but also for the control of bone turnover via secretion of receptor activator of NFκB ligand (RANKL), an essential cytokine for bone resorption by osteoclasts (10).

The process that links bone resorption to formation in a temporally and spatially coordinated manner within the remodeling cycle is called “coupling.” In order to maintain skeletal integrity, it is of crucial importance that the amount of bone resorbed exactly matches the amount of newly formed bone in each remodeling site. A negative bone balance over a longer period of time invariably leads to bone loss and osteoporosis, because a substantial amount of the skeleton is replaced each year in adult humans.

Numerous attempts have been made to explain how the information about the amount of bone resorbed by osteoclasts is transmitted to osteoblasts in the remodeling cycle. It is currently thought that coupling between bone resorption and formation occurs (i) through growth factors stored in the bone matrix, and released during resorption, (ii) through soluble clastokines secreted by osteoclasts, and (iii) through molecules expressed in the cell membrane of osteoclasts [reviewed by Sims and Martin (7)]. Most of our current understanding of the mechanisms involved in coupling comes from experiments in gene-targeted mice. However, mice and rats lack true intracortical, Haversian remodeling (1, 11). Therefore, it is unknown whether there are differences in the coupling mechanisms between intracortical and cancellous bone remodeling.

The current explanatory models of the coupling mechanism are associated with a number of problems. First, none of these models can convincingly explain why the amount of bone formed during the formation phase matches the amount of bone resorbed during the resorption phase. Second, and perhaps more critical is the fact, that in human cancellous bone remodeling, the time span between the end of osteoclastic resorption and the initiation of bone formation is in the range of several weeks (12). Any biochemical signal linking bone resorption to bone formation will have dissipated during this long period of time. Therefore, it is unclear how information is actually transmitted from osteoclasts to osteoblasts. Moreover, a diligently conducted histomorphometric study in human iliac biopsies of patients with postmenopausal osteoporosis revealed a large percentage (~30%) of remodeling cycles that became arrested in the reversal phase (4), suggesting that formation is not always tightly coupled to resorption in cancellous bone remodeling in humans.

These problems led me to hypothesize that coupling in cancellous bone remodeling may simply be a mechanically controlled process within the newly formed bone package. This hypothesis is actually not totally new, because several aspects of it have been described earlier (1, 12–20). However, it is presented here as a synthesis of different elements and in a refined form, taking into account the microanatomy of newly built bone packages. Using finite element models, Huiskes et al. (15) and Smit & Burger (16) provided mathematical descriptions of cancellous bone remodeling and of the potential strain distributions around a resorption cavity in cancellous and cortical bone remodeling, respectively, and suggested that strains sensed by osteocytes within resorption cavities could account for subsequent activation of osteoclasts and osteoblasts. Later on, these theories were further extended by including mathematical models of fluid flow in the osteocyte canalicular system around the resorption tunnel (18), and by simulation models for osteoclast activity (19). These mathematical models may explain why osteoclastic bone resorption proceeds along the loading axis, and why different strain distributions within the resorption cavity may account for spatial differences in the activation of different cell types. However, the latter models did not explicitly address the key feature of the coupling phenomenon, namely that the amount of newly formed bone matches the amount of previously resorbed bone in a given remodeling site. Huiskes et al. (15) proposed that the magnitude of the bone formation stimulus generated by osteocytes located in the underlying bone matrix may determine the number of osteoblasts recruited, and, thus, the amount of bone formed during the formation phase.

The conceptual advance of the current hypothesis is that it provides a plausible and self-regulating mechanism for the control of re-filling of the resorption cavity in cancellous bone remodeling based on the microanatomy of newly formed bone packages. In this model (Figure 1), the resorption cavity created by osteoclasts results in mechanical weakening of the structural element, and, thus, in increased strain around the resorption cavity under constant loading conditions. The increased strain is detected by osteocytes in the underlying bone matrix. This part of the hypothesis is supported by finite element models of the strain distribution around resorption cavities (16). When the strain exceeds a certain threshold, the osteocytes initiate subsequent bone formation by secreting osteogenic signals through the canalicular network opened by osteoclastic bone resorption (Figure 1A). After osteoblastic bone formation has started, the newly built osteocyte–osteoblast network detects strain, because the underlying osteocyte canaliculi system is sealed by the cement line (Figure 1B). All previous mathematical models have not taken into account that the cement line disrupts osteocyte signaling and also canalicular fluid flow from the underlying bone matrix to the surface. In addition, newly formed bone is less mineralized and has, therefore, different material properties compared with the higher mineralized surrounding old bone. It is likely that the differences in material properties between old and new bone affect strain energy distributions within the newly formed bone package, and, thus, mechanosensing of matrix-embedded osteocytes. Once the mechanical strain within the newly built bone structural unit falls below a certain threshold, bone formation stops. Because wall thickness has to be controlled within a range of a few micrometer to achieve constant trabecular thickness and bone mass, the strain threshold when bone formation stops needs to include the, depending on the species, 3–15 μm wide unmineralized osteoid seam (Figure 1B).

**Figure 1:**
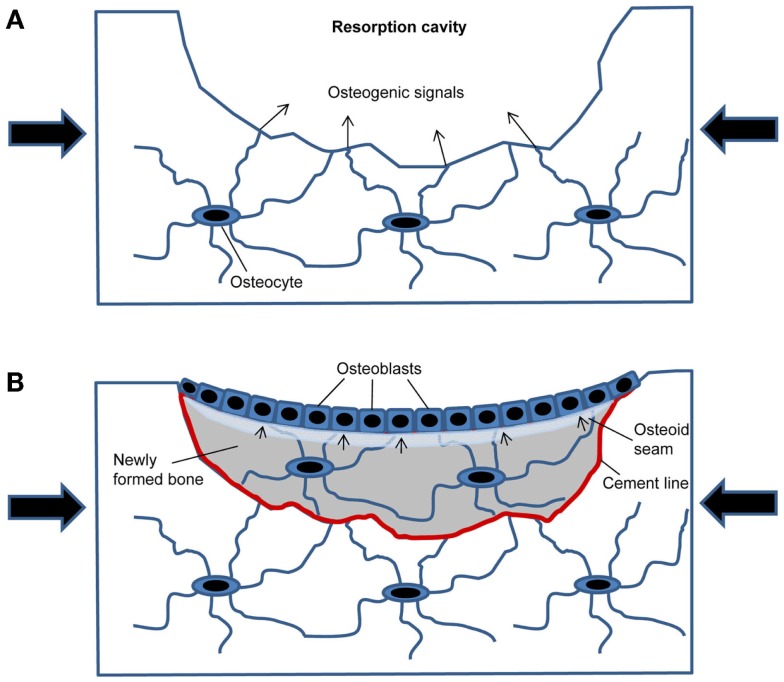
**Model of how mechanical strain within newly built bone packages induces coupling in cancellous bone remodeling**. **(A)** After osteoclastic bone resorption has been completed, the resorption cavity results in mechanical weakening and increased strain in the bone spicule when loaded (large arrows). Mechanosensing osteocytes detect the increased strain and secrete osteogenic factors to induce osteoblastic bone formation. **(B)** Filling of the resorption cavity is controlled by mechanosensing in osteocytes embedded within the newly formed bone, signaling (small arrows) to osteoblasts on the bone surface.

This model, which may also be used to further refine the mathematical models of bone remodeling, can explain why bone remodeling restores bone structures more or less in their old shape under unchanged loading conditions, i.e., in a biomechanical steady state. In this biomechanical strain-driven model, osteoblasts don’t need to “know” how much bone was previously resorbed in a given site. There is no necessity to transfer any information from bone-resorbing osteoclasts to bone-forming osteoblasts, because biomechanical strain within the newly formed bone package “guides” osteoblasts through their job of re-filling the resorption cavity. Further, this model explains why arrest lines can occur in bone remodeling units. Remodeling unit-associated arrest lines are frequently found in human (21) and also rat (1) bone sections. Arrest lines are generated when osteoblasts temporarily stop and subsequently resume their bone-forming activity. Based on the proposed model, arrest lines indicate a change in mechanical loading during the formation phase of the remodeling cycle, so that strain transiently falls below the threshold to maintain bone formation.

The proposed model makes a number of predictions which could be used to verify or falsify the model. In line with earlier mathematical models (15, 16, 19), the current model predicts that unloading will result in aborted remodeling and accumulation of resorption cavities. In addition, reduced biomechanical loading would lead to under-filling of resorption cavities. Conversely, increased loading would result in over-filling of resorption cavities. Moreover, shallow resorption cavities may not be filled with new bone by osteoblasts, because the increase in biomechanical strain of the structural element caused by a shallow resorption cavity may not be sufficient to elicit an osteogenic signal by osteocytes. In addition, mechanical disconnection of a structural element by excessive resorption and subsequent complete perforation will cause aborted remodeling, and changes in the material properties (e.g., hypo- or hyper-mineralization) of the newly formed bone will affect wall thickness.

Another interesting aspect of this model is that it could be regarded as a “unifying hypothesis of cancellous bone turnover.” It has long been an enigma why cancellous bone modeling and remodeling activities can coexist in a cancellous bone network, and how bone cells differentiate between these two different activities. In agreement with mathematical models reported previously (15, 19), the proposed model suggests that both processes follow the same rules and are just different aspects of the same underlying mechanism. For example, when strain falls below a certain threshold in a given structural element, parts of this element will be removed without subsequent induction of bone formation, resulting in a modeling resorption drift. Re-loading of the same element will induce formation on top of resorption which would be interpreted as remodeling in a histological section. The idea of a strain threshold for initiation of bone formation may also explain the observed lag time between the end of osteoclastic resorption and the initiation of bone formation (12). Especially in individuals with low levels of physical activity, it may take time to maintain strains above the threshold over a certain period of time in a specific remodeling site. The proposed model would predict that the lag time between resorption and formation depends on the biomechanical strain within a given structural element, and should be less in a high strain environment. In addition, it is possible that the thresholds may be modulated by endocrine signals (*vide infra*).

Is there any evidence for the validity of this model? In fact, there is. In a scanning electron microscopic study in lumbar vertebrae of normal subjects of different ages, Mosekilde (13) observed that resorption cavities were not filled with new bone on trabecule which lost 3D connection, i.e., unloaded trabecule. Moreover, partial unloading decreased mineral apposition rate, bone formation rate, and wall thickness in the presence of unchanged osteoclast numbers in a rat hindlimb immobilization model (Figure 2). All these experimental findings are predicted by the proposed model.

**Figure 2:**
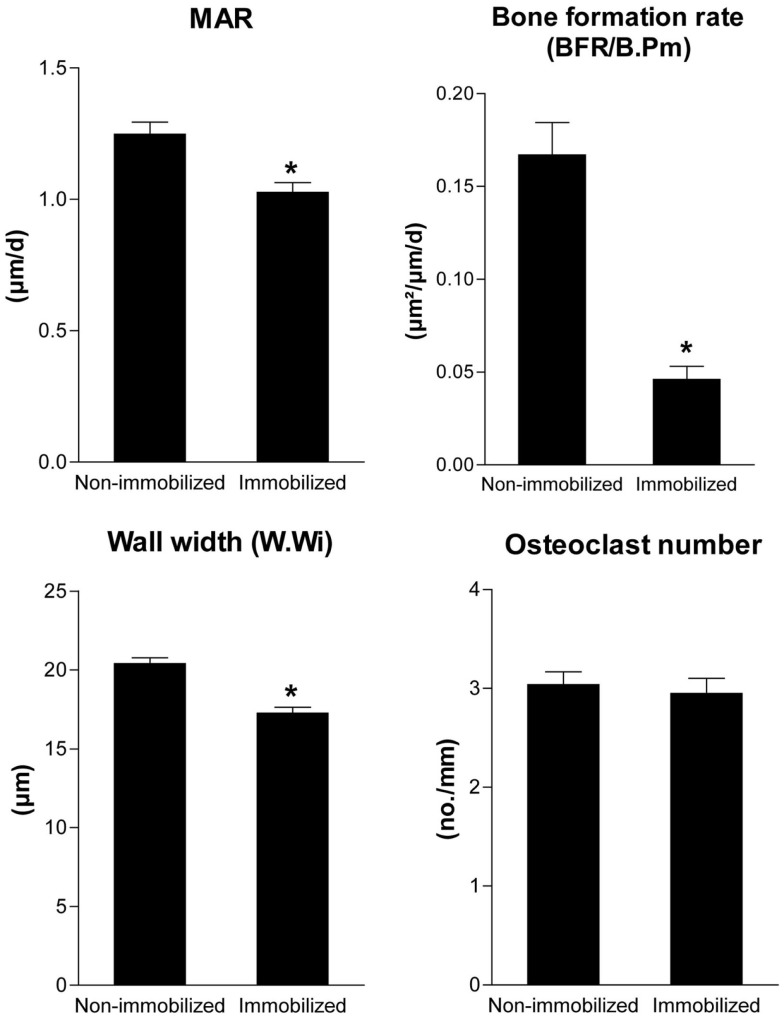
**Unloading reduces bone formation and wall width in the presence of unchanged osteoclast numbers**. Mineral apposition rate, bone formation rate (BFR/B.Pm), wall width of completed remodeling units, and osteoclast numbers measured by histomorphometry in the cancellous bone of the proximal tibial metaphysis in 4-month-old non-immobilized control rats and immobilized female Sprague-Dawley rats after 4 weeks of partial unloading by a bandaging technique (Erben et al., unpublished data). Data represent mean ± SEM of 12 animals each. *p < 0.05 by*t*-test.

The current hypothesis is dealing with the larger perspective of the physiological regulatory mechanisms underlying the coupling mechanism in cancellous bone remodeling, not with the actual biochemical signals involved in cell–cell communication. The nature of these biochemical signals is still controversial. However, it is highly likely that the Wnt pathway plays an important role for the osteocyte- or lining cell-driven initiation of bone formation in the proposed model. For example, treatment of rats and monkeys with anti-sclerostin antibodies induces modeling drifts on quiescent bone surfaces together with overfilling of remodeling units, thus, mimicking the effects of biomechanical over-loading (22). Therefore, the strain-induced downregulation of the Wnt inhibitors sclerostin or dickkopf-1 may be an important part of the physiological signaling mechanism in coupling. Thus, the nature of the “osteogenic” signal could in fact be a downregulation of inhibitory signals. Interestingly, mathematical models assuming that mechanical loading inhibits osteocytes from inhibiting bone formation via sclerostin secretion have been shown to be able to produce a load-aligned trabecular structure (23). It is also conceivable that the osteocyte-derived coupling signals are subject to hormonal modulation. For example, tight coupling was observed in patients with primary hyperparathyroidism (3). In this context, it is known that intermittent parathyroid hormone treatment suppresses sclerostin expression in bone (24). Therefore, it is possible that endocrine signals are able to modulate the osteocytic secretion of coupling factors.

The provocative aspect of the proposed model is that it challenges the notion of hemiosteonal remodeling with pre-determined BMUs in cancellous bone remodeling. Similar to strain-driven mathematical models of bone remodeling reported earlier (15, 16, 19), osteoclastic bone resorption and osteoblastic bone formation are not directly associated, but rather indirectly linked through mechanosensing osteocytes in the proposed model.

Whether loading might also influence the re-filling of resorption tunnels in Haversian remodeling in cortical bone is currently unknown. The same principles may apply, and mathematical models have been able to modulate both cancellous and intracortical bone remodeling, using similar parameters (19). However, an important difference between cancellous and cortical bone remodeling is that re-filling of the resorption tunnel in cortical bone remodeling is more or less complete, just leaving the Haversian canal in the center of the osteon. Unlike cancellous bone remodeling, the concentric contraction of the bone-forming surface during the formation phase in Haversian remodeling may simply result in mutual steric inhibition of bone-forming osteoblasts, so that bone formation automatically comes to a halt. Because there is no evidence that osteoblasts can move, most of the osteoblasts at a given position of the closing cone must undergo apoptosis. Therefore, theoretically a self-regulating process controlling the amount of bone formed by osteoblasts in the closing cone would not be necessary in Haversian remodeling. It might be enough just to initiate the process by the release of strain-dependent osteogenic signals after osteoclastic bone resorption. On the other hand, based on the concepts presented here for cancellous bone, strains sensed within the newly built bone matrix might also be necessary for maintenance of bone formation and complete re-filling of resorption tunnels in Harversian remodeling. This notion is supported by an earlier study in non-human primates, showing that transient unloading inhibited intracortical bone formation (25). Similar to cancellous bone remodeling, the lower degree of mineralization of the newly built bone matrix compared with the surrounding cortical bone might influence mechanosensing of osteocytes within the closing cone. Clearly, stringent experiments need to be designed to prove or disprove the proposed model of coupling in cancellous and possibly cortical bone remodeling.

## Conflict of Interest Statement

The author declares that the research was conducted in the absence of any commercial or financial relationships that could be construed as a potential conflict of interest.
